# An Account of a Case of Hydrophobia Successfully Treated by Copious Bleeding and Mercury. In Two Letters from Dr. Robert Burton, of Bent, in the State of Virginia, to Dr. Benjamin Rush, of Philadelphia

**Published:** 1805-08-01

**Authors:** 


### An Account of a Case of Hydrophobia successfully treated by copious Bleeding and Mercury. *In Two Letters from* Dr. Robert Burton, *of Bent, in the State of Virginia, to* Dr. Benjamin Rush, *of Philadelphia.*

[ Extracted from the American Medical Repository. ]

Sir,

Believing that you are always disposed to encourage any thing which may throw light upon the treatment of diseases, I take the liberty of addressing to you the following case of Hydrophobia, requesting a line or two, if you think it deserving your attention.

On July 4, 1803, at nine o'clock in the evening, I was desired to visit Thomas Brothers, aged twenty-eight years. I was informed by the person who came for me, that he had been bitten by a dog, which his friends suspected to be mad. I found him in the hands of four young men, who were endeavouring to confine him, and thereby prevent him from injuring himself or friends. He recognized me, and requested me to give him my hand, which he made a violent effort to draw within his mouth. Conscious of his inclination to bite, he advised his friends to keep at a distance, mentioning that a mad dog had bitten him.

His

His symptoms were as follow, viz. a dull pain in his head, watery eyes, dull aspect, stricture and heaviness at the breast, and a high fever.

Believing, as you do, that there is but one fever, I determined to treat this case as an inflammatory fever. I therefore drew twenty ounces of blood; and, as he refused to take any thing aqueous, I had him drenched with a large dose of calomel and jalap.

July 5th, four A. M. Finding the symptoms worse, I took away sixteen ounces of blood, and applied two large epispastic plasters to his legs, hoping thereby to relieve the oppression of the præcordia and other symptoms.

Twelve M. Was informed that one of his friends had permitted him to take a stick in his mouth, which he bit so as to loosen several of his teeth. As he craved something to bite, I desired his friend to give him a piece of lead, which he bit until he almost exhausted his strength.

One P. M. Finding but little alteration, I drew eighteen ounces of blood, and had him drenched with the antimonial powders.

Two P. M. He slept until half after three, when he awoke, with the disposition to bite, oppression, &c. but not so violent.

July 6th, eight A. M. Found him biting the bed-cloths; his countenance maniacal, his pulse synocha, with a stricture of the breast, difficult deglutition, laborious breathing, and a discharge of saliva. I took away twenty-four ounces of blood, gave him a dose of calomel and jalap, and continued the powders.

Twelve M. Drew sixteen ounces of blood, and gave him laudanum.

Five P. M. Found him in a slumber; his skin moist, and his fever and other symptoms much abated.

July 7th, eight A. M. Was informed that he had only two paroxysms during my absence, and that he had lost sixteen ounces of blood agreeably to directions. Notwithstanding the favourable aspect which the disease wore, I resolved to bleed him twice more, and then to induce an artificial fever by mercury, which would predominate over the hydrophobic. I therefore drew ten ounces of blood, and requested his friend to take eighteen ounces at night; to rub in a small quantity of mercurial ointment, and to give a mercurial pill every four hours.

July 8th, nine A. M. Found him convalescent, but continued the mercurial unction and pills.

July

July 9th, ten A. M. Found his gums sore, and discontinued the mercury.

July 15th, one P. M. Found him well, but with a considerable degree of debility.

It would be doing injustice to you not to mention that I was indebted to your lectures for the successful treatment of this disease.

*August* 21, 1803.

---

To Dr. BURTON,

Dear Sir,

Accept of my congratulations upon your rare triumph over a case of hydrophobia. I give you great credit for the boldness of your practice. You have deserved well of the profession of medicine.

In order to render your communication more satisfactory, permit me to request your answer to the following questions.

1. On what part of the body of your patient was the wound inflicted; and how long was the interval between the time of his being bitten and the attack of his fever?

2. Did he discover any aversion from the sight of water? and did he refuse to swallow liquids of all kinds?

3. What were the appearances of the blood drawn? Did it differ in the different stages of the disease?

Your answer to the above questions will much oblige your sincere friend,

*Philadelphia, Aug.* 29, 1808. BENJAMIN RUSH.

---

To Dr. BENJAMIN RUSH.

Sir,

I regret that business of an indispensable nature prevented me from being more particular in my communication. I drew it up in a hurry, intending to transcribe it, and insert such other notes as would throw light on the case; but being called out a few hours before the post set out from this place, I was obliged to forward the communication in the manner in which you received it.

The part of the body of my patient on which the wound was inflicted was a little above the union of the solæus and gastrocnemius muscles, which form the tendo-achillis. The interval between the time of his being bitten and the attack of the fever was twenty-four days.

He

He was, I was told, dull and solitary a few days previous to the attack. A few minutes before it his friends found him two hundred yards from the house, apparently in a deep study. He has informed me, since his recovery, that he had a slight pain in the wound, attended with itching, and an uneasiness in the inguinal gland, several days before the fever.

He refused to swallow liquids, and the sight of water threw him into a convulsive agitation.

With regard to the appearances of the blood drawn, I am sorry to inform you, that after it became cold I did not examine it. I am, Sir, yours, &c.

*Bent Creek, Virginia, Sept.* 18, 1803. ROBERT BURTON.

